# The London Hospital

**Published:** 1908-02-01

**Authors:** 


### HOSPITAL ADMINISTRATION.

### CONSTRUCTION AND ECONOMICS.

### THE LONDON HOSPITAL.

#### I. ITS DEVELOPMENT AND PRESENT CONDITION. SOME HISTORICAL FACTS.

We have known the London Hospital for forty years. We first inspected it as it then was in the year 1868, and we have recently spent a long day in going through its various divisions, studying its system and making ourselves familiar with the various departments and their actual condition and work as they exist to-day. We have often advocated the practical utility of writing and publishing a history of every hospital in the country which has had an existence of one hundred years or upwards. Not only could such histories be made most interesting in themselves, but they must contain material facts which would have a pecuniary value from the circumstance that they would help to bring home to the public the enormous developments which have taken place, and the vast improvements which have been made in our voluntary hospitals during the last forty years. Mr. Morris, the able and indefatigable secretary of the London, has made his own office one of the most interesting of secretarial workshops, by collecting together accurate and full plans of the actual condition of the buildings of the London hospital from the year 1759 to the present day. A careful study of them is in every sense an education in itself. This gallery of hospital plans enables the expert to follow with infinite clearness, and without loss of time, all the main incidents which have characterised the development of the modern hospital. They show, for instance, that in 1759 the ideas of what properly constituted a hospital building were most unhygienic, crude and improper.

The original London Hospital was built on the H plan, and the corridor at the end of each ward, nearest to the entrance, was boxed in, and contained three nurses' compartments, apparently without light or air, or even a window, which were placed at the back of the ward wall forming one side of the corridor. Opposite to them, in the same corridor, were three large open sinks which were presumably made of lead, into which everything appears to have been poured, and where all the washing up and necessary cleansing of utensils took place. The hospital at that time contained one cold bath. It also had a bleeding room. The secretary's office was placed upstairs, by no means a convenient arrangement, but the secretary in those days, being a clergyman, was also chaplain to the hospital. A piece of ground at one side of the ward was used as a burial ground. The secretary-chaplain seems to have had rather a gruesome idea of his business, for the minute book shows that he was instructed to discontinue the practice of reading the burial service over a number of bodies at once, and to bury one body at a time, with a separate service. One of the porters received sixpence for each grave he dug, and the hospital made a strong point of the fact, in appealing for funds, that the patients and their friends were put to no expense whatever when inmates of the hospital, and, should they die, they would be buried free of charge. It was a rule of the hospital in those days that each patient who was able to get up should attend every funeral. People sometimes speak of the inhumanity of the nineteenth century, but something might evidently be said of the peculiar ideas held by our forbears and sires in this respect, as typified by the methods of the London Hospital in 1759. These arrangements appear to have continued for seventy years, that is, until 1830, when an embryo medical school building was added to the left of the hospital buildings, with post-mortem and pathological rooms. These communicated with the right or west wing. The wards were constructed on the back to back principle, and when further accommodation was needed for patients in 1840 these old-fashioned wards were elongated and extended, and, as some of the burial ground was required for the purposes of building, it was moved farther back behind the hospital buildings proper. Up to sixty years ago all the nurses were recruited from outside, and none of them appear to have been resident in the hospital. There were no night nurses in the modern sense, but night watchers were engaged who received the large emolument of £5 a year for their services! We mention these interesting historical facts as indicating the condition of most of the voluntary hospitals in England for one hundred years at least prior to A.D. 1850.

In 1854 a new medical college was built, which indicates that at that time the medical school was recognised as forming an important part of the hospital, and that it was growing in importance and in the numbers of the students who entered for instruction. In 1866 the Alexandra Wing was added. It consisted of three wards, committee-room, house-governor's offices, with nurses' room on the upper floor. This latter fact indicates that at that time, *i.e.* in 1866, resident nurses were recognised as essential to the efficient working of the institution. In 1876 the Grocers' Wing was built. This was a most important addition in every sense of the word, for it provided improved wards, a new post-mortem department, indicating the growing importance of pathology in the opinion of the medical staff and the profession generally, and a small nurses' home, the first of its kind in connection with the institution. There was also an isolation block, but according to modern notions of hygiene it would scarcely have passed muster in the present day. Ten years later (1886) a chaplain's house was built; an enlarged and relatively fine nurses' home was added; the medical college was extended, and, even more important on economical grounds, embryo workshops were provided to enable the employment by the hospital of their own staff of artisans to execute the necessary repairs and to keep the whole establishment efficient. In 1897, when Mr. Holland became chairman, a second nurses' home was erected and an important addition was made by the erection of a handsome façade to the front of the hospital, and the provision of a covered entrance communicating with the Whitechapel Road. These changes involved a reconstruction of what was known as the central block, and they included a chapel, now abolished with the exception of a small oratory where a communion sarvice can be held, as the patients and staff now use St. Philip's Church as their chapel, a really great improvement, which should have a material effect for good on the whole tone of the institution.

Between 1901 and 1907 Mr. Holland moved his committee successfully to undertake a huge scheme of reconstruction which involved practically the rebuilding of the institution. It included the addition of the following new buildings, which cost a mint of money, as we have already explained in an article which appeared in The Hospital of August 17, 1907. These buildings included the new out-patient department, of which it may be said that, although 1,200 to 1,500 patients are seen each day in this building, the hygienic condition of the atmosphere when the work is at its highest is surprisingly good, and reflects credit upon those who are responsible for the ventilation and general design of the building. The Medical College was again enlarged; a new isolation block up to modern requirements was erected, and it is at the present time equipped and working under intelligent supervision, so that there is every prospect that the mass of valuable material available each year will be utilised to the full in the interests of medical science and the general welfare of the community. A third nurses' home, one of the largest in the country, was erected, which contains 273 bedrooms. A new laundry was put up, which is big enough to meet the whole of the growing requirements of the largest hospital in the country. The mere enumeration of these various matters, though they do not include by any means the whole of the work actually carried out, must indicate clearly to all intelligent people how vast is the work and how pressing are the claims of the London Hospital, the great general hospital of the east end of the metropolis.

It may be asked, Has the money produced all the results which were expected from its expenditure? No doubt if the work had to be done again, in the experience of recent years, the whole building would have been demolished, and an entirely new hospital erected from special plans on modern lines. However, as the London Hospital is to-day, we found it wonderfully complete and efficient. No one who is interested in hospitals should fail to pay it a visit, for an inspection of its many departments and the whole of its arrangements from top to bottom cannot fail to impress the expert inquirer, and indeed anyone who has hospital knowledge.

A big hospital should have its affairs conducted with some regard to perspective, and we take it that the training of medical students and the training of nurses at the London Hospital has this advantage—that it must broaden their minds and enable them to gain a wider grasp of the principles of modern hospital administration and also of its actualities than is ever possible in a small institution. Taking the old back-to-back wards, for instance, and examining them as they are to-day, it is evident that the best has been made of them in the scheme of reconstruction, that in many respects they are more comfortable to the patients than modern wards, and that hygienically they get the utmost possible circulation of air. We were told that the cases do exceedingly well in these wards, and also in the narrower children's wards, which have windows on one side only. These facts prove the efficiency of the management. Whatever opinion may be held as to the hygienic completeness of the London wards in the modern sense, no one who inspects the institution, can deny that the administration of the whole establishment is so thoroughly well done that the speedy return of a patient to health is quite readily accounted for. Again, the out-patient department building, though it has the disadvantage from an administrative point of view of being divided up into numerous relatively small departments upstairs and down, has been thoroughly well thought-out in an administrative sense, and smooth working is thus secured. On the other hand, the general supervision of such a building must prove relatively difficult. The lying-in department is a model in many respects, and we were struck by the high state of efficiency which it represents.

Some idea of the magnitude of the London Hospital may be gathered from the statement that there are no fewer than twelve operating theatres in the buildings devoted to in-patients, and that four more theatres are provided in the out-patients' building. We were interested to notice that the old lights, which were enormously costly, and which were at one time looked upon with disfavour, have been found by experience to produce the best results in the Finsen Light department, where they are kept actively at work for very many hours during each day of the week.

Taken altogether, having regard to its size and the fact that the buildings have been put up from time to time without necessarily any regard to the general plan of a big modern hospital, and realising the enormous difficulties which must attend the management of such a hospital as the London, we feel it is just to say that the efficiency attained in each department is exceedingly high, and that the moral effect of the discipline and system upon both the staff and the patients seems to be admirable. The reconstruction and extension of the London Hospital was a very difficult and gigantic task. It took a great amount of courage and energy to face it, and its successful accomplishment should win the confidence of the subscribers, who should make it their business to provide all the money necessary for the proper upkeep and maintenance of this vast establishment without hesitation and without delay.

Next week we propose to show how it should be possible to place the business side of the work of the London Hospital, once and for all, upon a sound financial basis.

